# Neuron-specific regulation of class I PI3K catalytic subunits and their dysfunction in brain disorders

**DOI:** 10.3389/fnmol.2014.00012

**Published:** 2014-02-13

**Authors:** Christina Gross, Gary J. Bassell

**Affiliations:** ^1^Department of Cell Biology, Emory University School of MedicineAtlanta, GA, USA; ^2^Center for Translational Social Neuroscience, Emory University School of MedicineAtlanta, GA, USA; ^3^Department of Neurology, Emory University School of MedicineAtlanta, GA, USA

**Keywords:** PI3K signaling, neuronal signal transduction, autism, epilepsy, schizophrenia

## Abstract

The phosphoinositide 3-kinase (PI3K) complex plays important roles in virtually all cells of the body. The enzymatic activity of PI3K to phosphorylate phosphoinositides in the membrane is mediated by a group of catalytic and regulatory subunits. Among those, the class I catalytic subunits, p110α, p110β, p110γ, and p110δ, have recently drawn attention in the neuroscience field due to their specific dysregulation in diverse brain disorders. While in non-neuronal cells these catalytic subunits may have partially redundant functions, there is increasing evidence that in neurons their roles are more specialized, and confined to distinct receptor-dependent pathways. This review will summarize the emerging role of class I PI3K catalytic subunits in neurotransmitter-regulated neuronal signaling, and their dysfunction in a variety of neurological diseases, including fragile X syndrome, schizophrenia, and epilepsy. We will discuss recent literature describing the use of PI3K subunit-selective inhibitors to rescue brain disease-associated phenotypes in *in vitro* and animal models. These studies give rise to the exciting prospect that these drugs, originally designed for cancer treatment, may be repurposed as therapeutic drugs for brain disorders in the future.

## INTRODUCTION

Signaling through phosphoinositide 3-kinases (PI3Ks) has diverse roles in the human body, regulating essential functions such as cell growth, migration, differentiation and survival. PI3K signaling is important for adequate immune response (Okkenhaug, 2013), hematopoiesis (Polak and Buitenhuis, 2012), and organ growth (Shioi et al., 2000). Mutations in PI3K catalytic subunits were found in primary immune deficiencies (Angulo et al., 2013) and in different forms of human cancer, including leukemia (Samuels et al., 2004; Gutierrez et al., 2009). Apart from a role in dividing cells, PI3K activity is also a key regulator of neuronal function. PI3K signaling transduces signals from cell surface receptors to the Akt/mTOR pathway and is essential for synapse and dendritic spine development (Jaworski et al., 2005; Chan et al., 2010; Cuesto et al., 2011; Lee et al., 2011) and for enduring forms of synaptic plasticity underlying learning and memory (Sanna et al., 2002; Man et al., 2003; Opazo et al., 2003; Sui et al., 2008; Hoeffer and Klann, 2010). Therefore, it is not surprising that an increasing body of evidence suggests dysregulated PI3K activity and downstream signaling as a key contributor and potential therapeutic target for mental disorders (Kalkman, 2006; Levitt and Campbell, 2009; Karam et al., 2010; Waite and Eickholt, 2010; Krueger et al., 2013).

### SPLITTING THE WORK – NEURONAL PI3K ACTIVITY IS MEDIATED BY SEVERAL CATALYTIC SUBUNITS

In vertebrates, PI3K enzymatic activity is brought about by eight different catalytic subunits. These catalytic subunits are divided into class I, class II, and class III PI3K enzymes according to their protein structure, function and associated regulatory subunits (Hawkins et al., 2006). Here, we will focus on class I PI3K catalytic subunits, which are further sub-divided into class IA and IB. The class IA isoforms, p110α (*PIK3CA*), p110β (*PIK3CB*), and p110δ (*PIK3CD*), are associated with any one of the following regulatory (inhibitory) subunits, which are encoded by three different genes: p50α, p55α, p85α (*PIK3R1*); p85β (*PIK3R2*) and p55γ (*PIK3R3*). In contrast, the (sole) class IB subunit p110γ (*PIK3CG*) associates with p101 (*PIK3R5*) or p87 (a.k.a. p84, *PIK3R6*). Class I PI3Ks predominantly function as lipid kinases and catalyze the phosphorylation of the third hydroxyl group of the inositol ring of phosphatidylinositol (PI), PtdIns-4-phosphate (PI(4)P), and PtdIns-4,5-biphosphate (PI(4,5)P_2_). The PI3K products PI(3,4)P_2_ and PI(3,4,5)P_3_ recruit proteins that contain pleckstrin homology (PH) domains to the membrane, leading to their activation (Lemmon, 2007). These PI3K-regulated proteins can have diverse functions, for example as signal transduction molecules, including protein kinases and GTPase-modifying enzymes (Rodrigues et al., 2000; Fayard et al., 2010).

There are two major modes of activation of class I catalytic PI3K subunits by extracellular stimuli, namely via receptor tyrosine kinases (RTKs) and via G protein-coupled receptors (GPCRs). Activation of p110 subunits via RTKs is mediated through interaction of the SH2-domain of the regulatory subunits (e.g., p85a/β) with a phospho-tyrosine on the C-terminal tail of the RTKs or on RTK-associated proteins (Hawkins et al., 2006). Activation by GPCRs is mediated via heterotrimeric G-proteins or the scaffolding protein Homer and the PI3K enhancer PIKE-L (Rong et al., 2003; Hawkins et al., 2006). Association of p110 subunits with these receptors leads to their recruitment to the cell membrane where they are in close proximity to their substrates. Notably, the different p110 isoforms appear to have preferences for either one or the other type of receptor, implying isoform-specific PI3K activation (Guillermet-Guibert et al., 2008).

**FIGURE 1:**
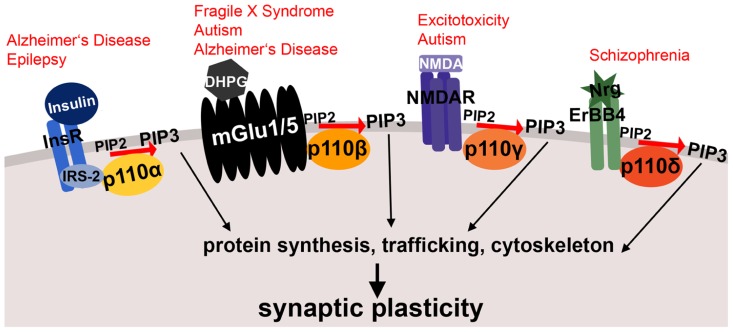
**Schematic illustrating membrane receptor-specific signaling of class I PI3K subunits and their potential link to mental disorders.** Shown are examples of neuronal membrane receptors and the specific p110 catalytic subunits, through which they preferentially signal. All of these receptor-PI3K complexes were implicated in mental disorders, which are printed in red above the receptors. Both insulin signaling through p110α and metabotropic glutamate receptor signaling through p110β are affected in Alzheimer’s disease. Moreover, metabotropic glutamate receptor signaling through p110β is altered in fragile X syndrome and other autism spectrum disorders. The NMDA receptor complex (associated with p110γ-selective activity) plays a role in excitotoxicity and epilepsy, and p110δ-mediated signaling through ErbB4 is dysregulated in schizophrenia. *InsR*, insulin receptor; *IRS-2*, insulin receptor substrate 2; *DHPG*, dihydroxyphenylglycine, mGlu1/5 agonist; *mGlu1/5*, metabotropic glutamate receptor 1/5; *NMDA*, N-Methyl-D-aspartic acid;* NMDAR*, NMDA receptor; *Nrg*, Neuregulin; *PIP2*, phosphatidylinositol-4,5-biphosphate; *PIP3*, phosphatidylinositol-3,4,5-triphosphate.**See text for references and further details.

Earlier reports suggested some functional redundancy between the class I isoforms, specifically in their ability to maintain cell proliferation (Foukas et al., 2010). However, later work in non-neuronal cells has shown that the p110 isoforms can have distinct cellular functions, and are signaling downstream of specific membrane receptors (Vanhaesebroeck et al., 2010). This observation led to the development of subunit-selective antagonists as therapeutics for cancer (Zhao and Vogt, 2008), which are currently tested in clinical trials (Akinleye et al., 2013). Most recently, p110 subunit-specific functions and mechanisms have begun to be discovered in the brain. The different p110 isoforms appear to have unique roles in mediating distinct forms of neuronal function and synaptic plasticity, suggesting the use of subunit-selective p110 inhibitors for certain brain disorders. The importance of PI3K catalytic subunit-selective roles in neurons is illustrated by functional and genetic studies that have linked dysregulation or mutations of specific p110 isoforms with distinct brain disorders. Given the essential function of PI3K signaling in non-neuronal cells, a precise knowledge of the molecular mechanisms of neuron-specific PI3K enzyme regulation and dysregulation in disease is mandatory for the development of therapeutic strategies ameliorating brain disorders without compromising other essential functions of the body. Here, we will review and discuss recent progress and open questions in our understanding of how the specific class I PI3K catalytic isoforms p110α, p110β, p110γ, and p110δ are regulated in neurons and how their dysfunction might lead to mental diseases (summarized in **Table 1** and **Figure 1**).

**Table 1:** This table summarizes the current knowledge about neuron-specific signaling and function of class I PI3K catalytic subunits and lists available tools for their future study (transgenic mouse models and drugs).

	PI3K subunit *(gene symbol)*	Neuronal signaling pathway	Physiological function in the brain	Neurological disease	Transgenic mouse models	Antagonists
Class IA	**p110α**(*PIK3CA*)	*Insulin receptor*	*Insulin-dependent plasticity/LTD*	megalencephaly, hemimegalencephaly Riviere etal. (2012) Epilepsy *Alzheimer’s disease *	→knockout (not viable) Bi etal. (2002) →transgenes with cancer mutations Koren and Bentires-Alj (2013)	INK1117^a^ BYL719^a^ A66
	**p110β** *(PIK3CB)*	mGlu1/5 S6, protein synthesis *Rac, Rab5*	protein synthesis Gross and Bassell (2012)	FXS Gross etal. (2010) Autism Cusco etal. (2009) *Alzheimer’s disease*	→knockout (not viable) Bi etal. (2002) →conditional knockout (liver) Jia etal. (2008)	TGX-221^b^ GSK2636771^a^ AZD-6482^a^ AZD8186^a^
	**p110δ ***(PIK3CD)*	Nrg1/ErbB4 RhoA	axon outgrowth and regeneration in sensory neurons Eickholt etal. (2007)	Schizophrenia Law etal. (2012)	→knockout Jou etal. (2002) →kinase-negative transgene^b^ Okkenhaug etal. (2002)	CAL-101^a^ IC87114^b^ TGR 1202^a^ AMG319^a^ PIK-294
**Class IB**	**p110γ ***(PIK3CG)*	NMDA Rap1, p38 *PDE3B*	NMDA-LTD, behavioral flexibility Kim etal. (2011)	Autism Serajee etal. (2003) *Excitotoxicity/Brain ischemia/Epilepsy*	→knockout^b^ Sasaki etal. (2000) →kinase-negative transgene Patrucco etal. (2004)	AS-605240^b^ CZC24832

*Italics point out that there is only indirect evidence to support the indicated roles.*

*^a^has been or is currently being used in clinical trials (cancer)*

*^b^used to analyze neuronal phenotypes*

#### p110α – insulin signaling to epilepsy and cognitive decline?

Each of the class I catalytic subunits has unique molecular features. P110α distinguishes itself from the other class I catalytic subunits by the absence of cell-transforming activity when overexpressed (Kang et al., 2006). Nevertheless, the majority of cancer-associated mutations in class I PI3K catalytic subunits were identified in the coding region of p110α. These mutations activate the enzymatic function and lead to oncogenic transformation (Samuels et al., 2004). In contrast, no oncogenic mutation in any of the other class I PI3K subunits has been reported so far. Interestingly, mutations in the kinase domain that activate p110α do not have an effect on p110β (Zhao et al., 2005) further corroborating the different modes of regulation of p110 catalytic subunits.

The important function of p110α in the brain is illustrated by enzyme-activating mutations in the p110α gene, *PIK3CA* that are associated with megalencephalies and hemimegalencephalies. These brain malformations lead to increased brain growth, developmental delay and epilepsy (Lee et al., 2012; Riviere et al., 2012). The p110α subunit is mainly activated by RTKs, and was shown to be a key mediator of insulin signaling in the liver (Sopasakis et al., 2010). Inhibitors of p110α but not p110β block insulin signaling in cultured cells (Knight et al., 2006). In the brain, insulin is important for cell survival and energy metabolism, but is also essential for PI3K-mediated regulation of synapse development (Lee et al., 2011) and enduring forms of synaptic plasticity (Zhao and Alkon, 2001). A brief exposure to insulin can induce long-term depression (LTD) at CA1 synapses that depends on PI3K signaling (Huang et al., 2003, 2004). It will be interesting to investigate if this form of LTD is mediated by p110α activity, whether it stimulates protein synthesis and how it might be affected by epilepsy-associated mutations in *PIK3CA*. Interestingly, correcting imbalances in insulin levels was suggested as therapeutic strategy for certain forms of epilepsy (Kim et al., 2013). Moreover, early stages of Alzheimer’s disease (AD) show signs of insulin resistance (Bosco et al., 2011), and insulin treatment is currently tested as a therapy in AD (de la Monte, 2013; Freiherr et al., 2013). Considering the predominant role of p110α in insulin signaling, selective manipulation of p110α activity may be beneficial to treat epilepsy or ameliorate cognitive decline in AD (**Figure 1**).

#### p110β – GPCRs, neuronal protein synthesis and autism

The p110β catalytic subunit is the predominant subunit associated with GPCRs (Guillermet-Guibert et al., 2008). This puts it in the unique position of being a key regulator of, e.g., metabotropic glutamate receptor 1/5 (mGlu1/5)-dependent forms of plasticity and protein synthesis in the brain. Interestingly, the regulatory subunits p85α and p85β have only reduced inhibitory effect toward p110β compared to other p110 subunits (Dbouk et al., 2010). RTKs activate PI3K signaling by releasing p85α/β-mediated inhibition of p110 subunits; lack of inhibition of p110β by p85α/β may thus contribute to the diminished stimulation of p110β signaling by RTKs (Kurosu et al., 1997; Guillermet-Guibert et al., 2008). Relatively low levels of p85α/β-mediated suppression of p110β may also cause the unusually high basal activity of p110β compared to other class I PI3K subunits.

The lack of this p85α/β-mediated inhibitory regulatory mechanism to suppress p110β activity under basal conditions suggests that increasing p110β protein levels through elevated p110β mRNA translation would directly lead to enhanced PI3K activity. In line with this assumption, the controlled expression of p110β appears to be an important mode of regulating p110β activity and PI3K-mediated protein synthesis in brain. Agonist-induced mGlu1/5 activation in mouse cortical synaptic fractions leads to increases in p110β protein levels and PI3K activity, which correlates with the PI3K-dependent stimulation of protein synthesis (Gross et al., 2010). p110β mRNA associates with and is translationally regulated by the fragile X mental retardation protein (FMRP), which is deficient in fragile X syndrome (FXS), the most common form of inherited intellectual disability and monogenic cause of autism (Gross et al., 2010; Sharma et al., 2010; Darnell et al., 2011). PI3K activity and protein synthesis are altered in FXS, and FXS mouse models and patient cells have increased p110β protein levels, which contributes to the observed elevated PI3K activity, downstream signaling and protein synthesis, and thus neuronal dysfunctions. Moreover, a duplication in the gene locus of p110β, *PIK3CB*, most likely leading to enhanced p110β-mediated PI3K activity, has been associated with autism (Cusco et al., 2009), further supporting an essential role of p110β expression in neuronal function. A p110β-selective inhibitor reduced the elevated protein synthesis rates in FXS mice and FXS patient cells suggesting that p110β has a crucial function to control neuronal protein synthesis (Gross and Bassell, 2012), and may be a promising therapeutic target for FXS and other autism spectrum disorders. However, more work is needed to assess the role of p110β and other p110 subunits in neuronal protein synthesis regulation and how this may be altered in human disease.

Defects in mGlu1/5-mediated signaling have not only been shown in FXS and other autism spectrum disorders (Williams, 2012), but also recently in AD (Ostapchenko et al., 2013; Um et al., 2013). The PI3K catalytic subunit p110β, similarly as discussed for p110α (*see above*), may thus also be a beneficial therapeutic target in certain forms of AD (**Figure 1**).

Signaling through p110β is unique, because it is not directly activated by the small GTPase Ras, as all other class I PI3K catalytic subunits (Zheng et al., 2012). Instead, it interacts with and is activated by Rac, a key regulator of the actin cytoskeleton (Fritsch et al., 2013), and by Rab5, a small GTPase essential for receptor-mediated endocytosis (Kurosu and Katada, 2001). The specific functions of Rac- and Rab5-mediated activation of p110β in neurons are unknown.

The phosphatase and tensin homologue (PTEN), a negative regulator of PI3K activity, which de-phosphorylates PI(3,4,5)P_3,_ was shown to preferentially bind to p110β compared to other PI3K catalytic subunits in non-neuronal cells. P110β is thus a key treatment target in cancers associated with PTEN mutations (Shepherd and Denny, 2012). Of note, PTEN loss-of-function mutations lead to autism (Zhou and Parada, 2012), and PTEN was shown to inhibit axonal regeneration in adult neurons (Park et al., 2008; Christie et al., 2010; Liu et al., 2010); however, the role of p110β-regulation of PTEN in brain function still remains to be discovered. Considering the predominant role of p110β downstream of GPCRs, it will be interesting if PTEN mutations in autism preferentially lead to impaired GPCR signaling, as opposed to other forms of plasticity.

#### P110γ – a key mediator of NMDA-dependent plasticity

The PI3K subunit p110γ is categorized as class IB due to the specific regulatory subunits it is associated with (p101 and p87), which are different from those associating with p110α, p110β, and p110δ. While p110γ has been shown to play a role in the immune system and the heart several years ago (Okkenhaug et al., 2002; Oudit and Kassiri, 2007), its functions in the brain have just recently begun to be discovered. Using *PIK3CG* knockout mice as well as a p110γ-selective inhibitor (Camps et al., 2005), Kim and colleagues showed the requirement of p110γ for establishing NMDA-dependent LTD in the CA1 region of the hippocampus (Kim et al., 2011; **Figure 1**). In contrast, other forms of long-term plasticity, such as long-term potentiation, as well as mGlu5-dependent LTD were not affected by p110γ deletion or inhibition. Moreover, a p110α-selective inhibitor, and a broad-spectrum class IA inhibitor both did not affect NMDA-LTD, strongly suggesting a unique role of p110γ in NMDA-LTD in the hippocampus. The physiological role of p110γ for neuronal function was further corroborated by the observation that p110γ deletion led to impairments in reversal learning in mice.

NMDA receptor-mediated excitotoxicity depends on PI3K signaling (Brennan-Minnella et al., 2013). In view of the study by Kim et al. (2011), it will be interesting to examine if p110γ is critical for excitotoxicity and thus may have therapeutic potential to prevent excitotoxic events in the brain (**Figure 1**). P110γ associates with and activates phosphodiesterase 3B (PDE3B) in the heart, leading to increased cAMP levels in its absence (Patrucco et al., 2004). PDE3B is expressed throughout the brain (Reinhardt and Bondy, 1996) and up-regulated in cortical astrocytes and neurons after ischemic insult (Mitome-Mishima et al., 2013), but the function of p110γ-mediated regulation of PDE3B in neurons is unknown.

Corroborating an essential role of p110γ for neuronal plasticity, there is also a genetic link between p110γ dysfunction and mental disorders, particularly autism. The *PIK3CG* gene is located within the autism susceptibility locus *AUTS1* on chromosome 7q22 (International Molecular Genetic Study of Autism Consortium, 2001; Kratz et al., 2002). Single nucleotide polymorphisms in *PIK3CG*, *TSC1*/2, which is mutated in the autism spectrum disorder tuberous sclerosis (TS), and *INPP1*, inositol polyphosphate-1-phosphatase, were shown to be in linkage disequilibrium in patients with autism (Serajee et al., 2003). This polymorphism was detected in the accessory domain (PIK domain) of p110γ, which is involved in substrate recognition (Domin and Waterfield, 1997). However, the polymorphism does not change the amino acid composition, and the effect it may have, e.g., on p110γ expression is unknown. Future work will have to show if p110γ dysregulation, either functional up- or down-regulation, can lead to autistic behavior in animal models.

#### P110δ – essential for developing axons and dysregulated in schizophrenia

The catalytic subunit p110δ was originally identified as key component of lymphocyte signaling (Okkenhaug, 2013) and a recent study reporting a specific enzyme-activating mutation in p110δ in humans with recurrent respiratory infections further supports an essential role of the p110δ subunit in the immune system (Angulo et al., 2013). In addition, a critical role for p110δ in neurons has become increasingly evident over the last years. A study using knockout mice and dominant negative forms of p110δ has shown that p110δ is essential for axonal outgrowth during development and in regenerating neurons (Eickholt et al., 2007).

More recently, increased p110δ mRNA expression and dysregulated p110δ-mediated signaling was associated with schizophrenia (Law et al., 2012), suggesting p110δ-selective inhibitors as a novel treatment strategy for schizophrenia and other psychotic diseases (Rico, 2012). Law and colleagues showed that p110δ is the major PI3K catalytic isoform signaling downstream of the neuregulin 1 (Nrg-1) receptor ErbB4 (Law et al., 2012; **Figure 1**). Both ErbB4, as well as Nrg-1 have been identified as risk genes for schizophrenia (Stefansson et al., 2002; Law et al., 2006; Norton et al., 2006; Silberberg et al., 2006). There are several isoforms of ErbB4, which have different capabilities of binding to, and activating PI3K catalytic subunits (Veikkolainen et al., 2011). Schizophrenia-associated polymorphisms lead to increased expression of the CYT-1 isoform of ErbB4, which is coupled to PI3K signaling (Law et al., 2007). These findings suggest dysregulation of the Nrg1-ErbB4-p110δ signaling complex as a risk factor for schizophrenia, and corroborate the importance of PI3K isoform-specific signaling mechanisms in neurons. It will be interesting to assess whether schizophrenia-associated mutations result in impairments in Nrg1-induced activation of p110δ-associated PI3K signaling and protein synthesis, suggesting parallels with p110β dysregulation in FXS.

Interestingly, ErbB4 was shown to be predominantly expressed in GABAergic interneurons in both the frontal cortex as well as the hippocampus (Vullhorst et al., 2009; Neddens et al., 2011). Using transgenic mice with cell type-specific ErbB4 deletions or overexpression, a recent study confirmed a major role of ErbB4 in dendritic spine morphology in parvalbumin-positive interneurons, but not pyramidal neurons (Yin et al., 2013). Studies in *Drosophila *corroborated the role of PI3K signaling in dendritic spine formation and synaptic plasticity in brain interneurons (Acebes et al., 2011, 2012); however, the role of p110δ or any other class I p110 subunit in vertebrate interneurons is unknown. To further elucidate the defects of ErbB4-p110δ signaling in schizophrenia it will be important to examine the specific roles of p110δ and other p110 isoforms in interneurons and other neuronal subtypes.

### CHALLENGES AND OPEN QUESTIONS

The discussed studies are most likely just the tip of the iceberg illustrating the diverse and unique functions of the different class I p110 isoforms in the brain. These mechanisms of specialized PI3K signaling and regulation add to the variety of tools neurons utilize to achieve circuit-, cell-, synapse-, and stimulus-specificity. Future challenges will be to understand how receptor complex-specificity of the different PI3K subunits is achieved, how they are regulated developmentally and whether there are cell type- or brain circuit-specific differences in isoform signaling, as implied in the case of ErbB4 and p110δ. In particular, it will be interesting if distinct p110 subunits are selective transducers of mTOR-mediated protein synthesis regulation by different receptors in neurons. Possible mechanisms of p110-regulation may include control of local translation [as suggested by the presence of p110β mRNA in neuronal dendrites (Gross et al., 2010)] or the generation of local micro-domains of PI3K signaling by receptor and scaffold clustering (Gao et al., 2011).

As mentioned throughout this review, class I PI3K catalytic subunits were shown to be dysregulated in various forms of mental disorders. They seem to play important roles in the disease phenotypes, as shown by the therapeutic effect of isoform-selective inhibitors in preclinical studies. The discussed examples for p110δ in schizophrenia and p110β in FXS provide models, which are corroborated in mice and human patient cells. In the future, it will be interesting to determine if the disease phenotypes caused by p110 dysfunction are unique to specific p110 isoforms or if defects in the same isoform can lead to different types of brain diseases.

PI3K activates the mTOR pathway, which has been shown to be dysregulated in autism spectrum disorders of different etiologies, and was suggested as a therapeutic target (Wang and Doering, 2013). Targeting mTOR is an alternative approach to p110 subunit-modulating drugs that might be advantageous in some cases, because it might correct defects in several upstream pathways impinging on mTOR. The utility of mTOR inhibitors for TS has been shown in a mouse model (Tsai et al., 2012). In TS, the effected protein complex, TSC1/TSC2, lies almost directly upstream of mTOR (Inoki et al., 2002). A potential disadvantage of targeting mTOR is that it plays a crucial role in protein synthesis regulation in many different receptor pathways. In contrast, the specific manipulation of single PI3K catalytic subunits has the potential of being more selective to the receptor pathway that is primarily effected, and thus disease-targeted, leading to enhanced efficacy (**Figure 1**, **Table 1**). In the future, it will be interesting to investigate if mTOR is equally activated by all p110 subunits, or if specific p110 isoforms play more important roles than others, which could aid the development of future therapeutic strategies targeting mental disorders with impairments in mTOR.

Subunit-selective inhibitors potentially represent powerful therapeutic tools as they should not have deleterious effects on global PI3K activity, but rather only achieve selective inhibition of PI3K-activity coupled to specific receptors. Future research on the involvement of specific receptor-associated PI3K-signaling complexes may thus lead to the development of novel therapeutic strategies for autism, epilepsy or schizophrenia.

## Conflict of Interest Statement

The authors are co-inventors on patent application PCT/US2010/055387, which suggests the use of (1) PI3K antagonists as a therapeutic treatment for fragile X syndrome and other autism spectrum disorders and (2) PI3K activity as a biomarker for these diseases.
